# Small-Pox in Germany

**Published:** 1904-04-30

**Authors:** 


### The Hospital.

A Journal of

The Medical Sciences and Hospital Administration.

Vol. XXXVI.—No. 918. APRIL 30, 1904.

### SMALL-POX IN GERMANY.

A favourite contention of anti-vaccinists has just received its death-blow at the hands of the Local Government Board. The immunity of Germany from small-pox epidemics, notwithstanding the frequency with which the disease is brought over the frontiers from Russia, Austria, and Italy, has long been a noteworthy and conspicuous fact; and, as it dates from the period at which the revaccination law of 1874 came into force, it has been ascribed, by most people possessing the rudiments of common sense, to the immunity thus conferred upon the population as a whole. The anti-vaccinists in this country, however, have not been content to accept this simple and obvious explanation; but have volubly maintained that the immunity in question is due to the completeness of the German isolation of occurring cases, and that it might be obtained in an equal degree by the enforcement of similar isolation among ourselves. In order to determine the point, and on account of its obvious bearing upon the future legislation of this country, the Local Government Board instructed Dr. Bruce Low, one of its inspectors whose work has largely lain among epidemics and their causes, to proceed to Germany and to investigate the facts at the fountain head; and the results of his inquiries have just been published in the form of a Parliamentary paper which may be obtained from any bookseller at the moderate price of one shilling, and which it would be a meritorious act on the part of the Imperial Vaccination League or any similar body to render even more generally and more cheaply accessible. Dr. Low went in the first instance to Berlin, to confer with the Central Imperial Health Office, but as no cases of small-pox, as far as the Office was aware, existed in Germany at the time of his visit, it was arranged that he should extend that visit to representative towns in the four chief States of the German Empire; namely, to Berlin, Cologne, Frankfort-on-Main, Weisbaden, and Mainz, in the kingdom of Prussia; Munich and Nuremberg, in the kingdom of Bavaria; Dresden and Leipzig, in the kingdom of Saxony; and Stuttgart, in the kingdom of Württemberg. In each of these towns he conferred with the physicians of the hospitals, except at Mainz, where circumstances compelled him to be content with the evidence of lay officials, inspected the accommodation provided for small-pox patients, and obtained general information from local medical men, municipal officials, and others.

Before proceeding to give the detailed results of his inquiry, Dr. Low explains that, under the law of 1874, and throughout the German Empire, young children must be vaccinated before the end of the calendar year following the year of birth, and all school children must be revaccinated in their twelfth year, obedience being enforced by fine or imprisonment. The notification of small-pox to the police authorities is obligatory throughout the Empire; and, as soon as these authorities have received a notification, the information is at once communicated to the medical officer of health, whose duty it is to examine into the circumstances and to report. All persons suffering or suspected to be suffering from small-pox can be ordered to hospital for isolation if, in the opinion of the medical adviser of the local authority, they cannot be isolated at home.

The most remarkable part of the case, however, is that in Germany, with a very few exceptions, there are no "small-pox hospitals," and there is nothing which would be called isolation according to the English acceptation of the term. The patients, as a rule, are removed to the general hospital of the town or district, where commonly a pavilion is kept in readiness for their reception, although it is often thought enough to clear out the patients from some occupied pavilion and transfer them elsewhere, to make room for the small-pox case or cases. At Frankfort-on-Main the portion of the general hospital site on which the small-pox pavilion stands is enclosed by a wall, but, in the absence of small-pox, this pavilion is used for the treatment of other maladies, infectious or not, as may be required. At Dresden arrangements are made to erect a temporary wooden fence round the small-pox pavilion as soon as a case is admitted. But, generally speaking, the cases are "isolated" in a pavilion standing on the site of the general hospital and in no way shut off from the rest of the establishment. In most cases this pavilion is supplied with food from the central kitchen, and the soiled linen is sent to the general laundry after being steeped in some disinfecting solution. The nurses and the medical officer or any other persons whose business brings them into relation with the small-pox pavilion are at once revaccinated as a matter of routine; but revaccination of the whole hospital *personnel* is not attempted, complete reliance being placed upon the statutory vaccination and revaccination of the whole people, as conducted under the law of 1874.

It follows that in Germany there is no isolation of small-pox as we should understand it, and that there is certainly nothing of the kind which would even hinder, much less prevent, the occurrence of widespread epidemics among a susceptible population. In England, as we know only too well, such epidemics not only occur, but are sources of great mortality, great suffering, and great expense. In Germany they are unknown, obviously because the people generally have been rendered insusceptible; although frequent outbreaks of the disease have occurred in towns in the vicinity of the frontiers and also in the persons of foreigners resident in or visiting Germany. As a general result there were in Germany, among a population of over fifty-six millions, 607 deaths from small-pox in the twelve years 1891-1902; while in England and Wales, during the same period, in a population of thirty-two and a-half millions, the deaths from the same cause amounted to 6,761.

